# CCL2 and CCL5 driven attraction of CD172a^+^ monocytic cells during an equine herpesvirus type 1 (EHV-1) infection in equine nasal mucosa and the impact of two migration inhibitors, rosiglitazone (RSG) and quinacrine (QC)

**DOI:** 10.1186/s13567-017-0419-4

**Published:** 2017-02-27

**Authors:** Jing Zhao, Katrien C. K. Poelaert, Jolien Van Cleemput, Hans J. Nauwynck

**Affiliations:** Laboratory of Virology, Department of Virology, Parasitology and Immunology, Faculty of Veterinary Medicine, Ghent University, Salisburylaan 133, 9820 Merelbeke, Belgium

## Abstract

Equine herpesvirus type 1 (EHV-1) causes respiratory disease, abortion and neurological disorders in horses. Besides epithelial cells, CD172a^+^ monocytic cells become infected with EHV-1 in the respiratory mucosa and transport the virus from the apical side of the epithelium to the lamina propria en route to the lymph and blood circulation. Whether CD172a^+^ monocytic cells are specifically recruited to the infection sites in order to pick up virus is unknown. In our study, equine nasal mucosa explants were inoculated with EHV-1 neurological strains 03P37 and 95P105 or the non-neurological strains 97P70 and 94P247 and the migration of monocytic cells was examined by immunofluorescence. Further, the role of monokines CCL2 and CCL5 was determined and the effect of migration inhibitors rosiglitazone (RSG) or quinacrine was analyzed. It was shown that with neurological strains but not with the non-neurological strains, CD172a^+^ cells specifically migrated towards EHV-1 infected regions and that CCL2 and CCL5 were involved. CCL2 started to be expressed in infected epithelial cells at 24 h post-incubation (hpi) and CCL5 at 48 hpi, which corresponded with the CD172a^+^ migration. RSG treatment of EHV-1-inoculated equine nasal mucosa had no effect on the virus replication in the epithelium, but decreased the migration of CD172a^+^ cells in the lamina propria. Overall, these findings bring new insights in the early pathogenesis of EHV-1 infections, illustrate differences between neurological and non-neurological strains and show the way for EHV-1 treatment.

**Electronic supplementary material:**

The online version of this article (doi:10.1186/s13567-017-0419-4) contains supplementary material, which is available to authorized users.

## Introduction

Equine herpesvirus 1 (EHV-1) is an important pathogen of horses. It is a member of the subfamily *Alphaherpesvirinae* with a 150 kb double stranded DNA genome [1]. Alphaherpesviruses of different species have developed in evolution various ways to reach deeper tissues of the upper respiratory tract in order to find lymph and blood vessels for further spread and neurons for inducing latency. Among them, pseudorabies virus (PRV), bovine herpesvirus-1 (BHV-1) and herpes simplex virus-1 (HSV-1) easily spread across the basement membrane (BM) in a plaquewise manner upon the activation of cellular proteases whereas EHV-1 employs a more discrete manner to invade [2–4]. It hitchhikes across the BM using local immune cells, mainly CD172a^+^ cells as Trojan horse [5]. EHV-1 enters CD172a^+^ cells via an endocytic mechanism that requires cholesterol, tyrosine kinase activity, actin, dynamin activity and endosomal acidification, pointing towards a phagocytic mechanism [6]. EHV-1 infection of nasal mucosa epithelial cells leads to an increase of the thickness of the collagen VII and a degradation of integrin alpha 6 of the BM underneath the EHV-1 plaques [7]. Afterwards, a cell-associated viremia allows EHV-1 to reach internal organs such as the pregnant uterus and/or central nervous system (CNS). Replication in these organs may result in abortion, neonatal foal death and myeloencephalopathy [8, 9]. Based on the difference of a single nucleotide polymorphism (A2254/G2254) in the EHV-1 DNA polymerase gene (ORF30), EHV-1 can be divided into neurological strains and non-neurological strains [10]. It has been reported that the neurological strains infect a higher number of CD172a^+^ cells than the abortigenic strains in the upper respiratory mucosa [5].

Our lab has found that CD172a^+^ monocytic cells can become infected with EHV-1 in the respiratory mucosa and transport the virus from the apical side of the epithelium to the lamina propria en route to the lymph and blood circulation [11]. In general, cytokines and chemokines are orchestrating the migration of monocytic cells during viral infections in the airways [12, 13]. It has been reported that infection of alveolar epithelial cells with influenza A virus can strongly induce the release of monocyte chemoattractants CCL2 and CCL5 followed by a strong recruitment of monocyte transepithelial migration [14]. Whether EHV-1 infection is activating the attraction of CD172a^+^ monocytic cells to the infection sites and whether CCL2 and CCL5 are driving forces during this process are largely unknown. In a previous study, it has been shown that EHV-1 infected PBMC can up-regulate inflammatory chemokines CCL5, CXCL9 and CXCL10, and down-regulate chemotactic CCL2 and CCL3 with clear strain differences [15]. During an infection with another alphaherpesvirus, HSV-1, in mice, it has been reported that CCL3 attracts NK cells [16], CCL2 recruits monocytes [17], and CCL5 recruits monocytes, NK cells, and PMNs [18] while CXCL9 recruits T-cells to the sites of infection [16, 19]. In our study, we mainly focused on the well-known monokines CCL2 and CCL5. IL-8/CXCL8 that can specifically induce neutrophil recruitment during an EHV-1 infection [20] was also included.

EHV-1 infection has a significant economical impact on the equine breeding industry worldwide every year [21]. Current vaccines do not provide full protection against severe symptoms induced by EHV-1 and there is no efficacious antiviral treatment available for EHV-1 infection. As CD172a^+^ cells function as Trojan horses during EHV-1 invasion in the respiratory mucosa, inhibition of the recruitment of these cells may prevent migration of infected monocytic cells into the deep tissues. This might be an effective way to impede the generation and invasion of infected CD172a^+^ cells and reduce the viremia. Phenotypical and functional analysis of the nasal mucosal CD172a^+^ cells indicated that they mainly consist of immature dendritic cells (DC) [22].

Thus, DC migration inhibitors might be an option to inhibit the EHV-1 deep invasion. It has been demonstrated that rosiglitazone (RSG), which has been used to treat type 2 diabetes, can specifically impair the departure of Langerhans cells (LCs) from the epidermis and moreover can block accumulation of DC in the draining lymph nodes (DLNs). In the respiratory mucosa, RSG can also inhibit the migration of DCs from the airway mucosa to the thoracic lymph nodes (LNs) [23]. Another DC migration inhibitor, quinacrine (QC), originally used as an antiprotozoal and anti-rheumatic agent, has also been reported to inhibit the epidermal DC migration by blocking NF-κB-dependent production of TNF-α, IL-1β and CCL21 in the skin [24].

In the current study, equine nasal mucosa explants were used as a model to study whether CD172a^+^ monocytic cells are specifically recruited to the EHV-1 infection sites in order to capture virus and whether the monokines CCL2 and CCL5 are involved during this process. In addition, treatment with RSG or QC at different concentrations was performed 12 h prior to or at the same time of the viral inoculation to test whether the treatment can impede EHV-1 deeper infection.

## Materials and methods

### Cells and virus

RK-13 cells were used. They were cultured in Dulbecco’s Modified Eagle Medium (DMEM) (Invitrogen, Paisley, UK) supplemented with 10% fetal calf serum (Invitrogen), 100 U/mL penicillin, 0.1 mg/mL streptomycin and 1 μg/mL gentamicin.

Four Belgian EHV-1 strains were used in this study. The neurovirulent strains 95P105 and 03P37 were originally isolated in 1995 and 2003 from the blood of a paralytic horse [25, 26] and the abortigenic strains 97P70 and 94P247 strains were isolated in 1997 and 1994 from the lungs of an aborted fetus [27]. All the EHV-1 strains were at the sixth passage, four passages in equine embryonic lung cells and two subsequent passages in RK-13 cells, with a titer of 10^6.5^ tissue culture infectious dose with 50% endpoint per milliliter (TCID_50_/mL). For virus inactivation, a thin layer of viral suspension was exposed to short-wave UV light at 1.2 × 10^5^ µJ/cm^2^ for 10 min. Absence of viral infectivity was checked by virus titration on RK-13 cells.

### Equine nasal mucosa explants and inoculation with EHV-1

The nasal mucosa explants were collected from 3 healthy horses, between 4 and 6 years old at a local slaughterhouse. Firstly, the nasal mucosa explants were stripped from the nasal surface and divided into pieces of 50 mm^2^. After 24 h of culture on fine-meshed gauze, explants were washed twice with warm medium and transferred on top of solid agarose. The margins were filled with agarose. Afterwards, the explants were inoculated with 500 μL medium containing 10^6.5^ TCID_50_ EHV-1 of the neurological strains 03P37 and 95P105 or the non-neurological (abortigenic) strains 97P70 and 94P247 for 1 h at 37 °C with 5% CO_2_, respectively. After inoculation, the explants were rinsed and further incubated with fresh medium [28]. Mock inoculations, incubated with DMEM medium, were performed in parallel.

### Localization and quantification of CD172a^+^ cells in equine nasal mucosa explants

**Figure 1:**
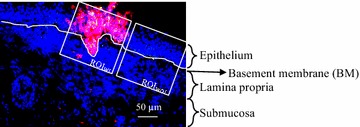
**EHV-1 neurological strain 03P37 infected equine nasal mucosa with two regions of interest.** ROI_WI_ is the region of interest including the epithelium and the lamina propria with EHV-1 infection in the epithelium whereas ROI_WOI_ is the region of interest without EHV-1 infection in the epithelium. The white line drawn on the image represents the basement membrane (BM). Scale bar: 50 µm.

At 0, 24, 48 and 72 h post-incubation (hpi) the explants were collected and snap frozen in methocel™. At each time point, 20 serial 16 µm cryosections were made for immunofluorescence stainings. In brief, biotinylated equine polyclonal anti-EHV-1 IgG (diluted 1:10 in PBS) [26], followed by streptavidin-Texas Red® (Invitrogen, 1:200 in PBS) were added to check for EHV-1 infected cells. Next, mouse monoclonal antibody (mAb) DH59B (VMRD Inc., Pullman) was used as cell marker to detect CD172a^+^ cells, followed by FITC-labeled goat anti mouse IgG. The nuclei were counterstained with Hoechst 33 342 (10 μg/mL, Molecular Probes). After staining, the cryosections were rinsed three times in PBS and mounted with glycerin/1,4-diazabicyclo [2.2.2] octane. All IF stainings were analyzed by confocal microscopy (Leica Microsystems GmbH, Wetzlar, Germany). An appropriate isotype-matched, irrelevant control (IgG1) mouse monoclonal anti-PRV gD antibody 13D12 was included for testing the specificity of the stainings [29]. Two regions of interest (ROI) were chosen for the quantification of CD172a^+^ cells and EHV-1^+^ cells. One was the region where the epithelial cells were infected with EHV-1 (ROI_WI_) and the other was the region without EHV-1 infection in the epithelial cells (ROI_WOI_) (Figure 1). Three independent replicates were performed and 20 images were taken for each experiment at each time point.

### The expression of chemokines CCL2 and CCL5 in EHV-1 infected nasal mucosa explants

To check whether chemokines CCL2 and CCL5 may be involved in the recruitment of CD172a^+^ cells towards the infected epithelial cells, their expression was examined. The equine nasal mucosa explants were collected, cultured and inoculated with EHV-1 as described above. Mock inoculations were carried out in parallel. A scratch-wound assay using a yellow pipette tip to make a straight scratch and simulate a wound was performed to detect the expression of CCL2 or CCL5 in the wounded nasal mucosa explants. Inoculation with UV-inactivated EHV-1 was performed to test whether the production of CCL2 or CCL5 could be induced only by the binding/entry step of EHV-1 to the epithelial cells. Finally, the explants were collected at 0, 24, 48 and 72 hpi, embedded in methocel™, and snap frozen at −70 °C.

Of each explant, 50 serial 16 µm cryosections were made for IF stainings and the aforementioned protocol was followed to detect EHV-1 infected cells. For the detection of CCL2 or CCL5, rabbit polyclonal anti-CCL2 IgG or anti-CCL5 IgG (Biorbyt, 1:100 in PBS) was used as primary antibody, followed by goat anti-rabbit IgG FITC (1:100) (Invitrogen, 1:100 in PBS), respectively. The rabbit polyclonal anti-CXCL8 IgG (Mybiosource, 1:100 in PBS) was used as control. All the results of IF stainings were analyzed by confocal microscopy. By using the software imaging system ImageJ, the percentage of pixels positive for CCL2 or CCL5 was determined. Two ROIs were chosen for the quantification of the expression of CCL2 or CCL5. One was the region where the epithelial cells were infected with EHV-1 (ROI_WI_) and the other was the region without EHV-1 infection in the epithelial cells (ROI_WOI_) (Figure 1). Three independent replicates were performed for each experiment.

### Evaluation of tissue toxicity of rosiglitazone (RSG) and quinacrine (QC) on nasal mucosa explants

As DC migration inhibitors might be useful to inhibit the deep invasion of EHV-1 in respiratory mucosa via infected monocytic cells, two DC migration inhibitors RSG or QC (Sigma-Aldrich) were used to treat equine nasal mucosa explants. TUNEL staining was performed to assess the tissue toxicity of RSG or QC (1, 3, 10, 30 μM) [23] on the nasal mucosa explants. Briefly, after 24 h culture on fine-meshed gauze, these explants were transferred into 24-well plate and immersed with RSG or QC at different concentrations (1, 3, 10, 30 μM) for 1 h at 37 °C with 5% CO_2_. Afterwards, the explants were transferred back to the gauze and cultured within the medium in the presence of RSG or QC at corresponding concentrations. Untreated explants were immersed in and incubated with medium in the absence of RSG or QC. At 0, 24, 48 and 72 hpi, the explants were collected and snap frozen in methocel™ at −70 °C. Cryosections were made and the TUNEL reaction was performed according to the manufacturer’s guidelines. TUNEL-positive cells were counted in five randomly chosen fields of 100 cells in the epithelium as well as in the lamina propria with confocal microscopy.

### The effect of RSG or QC on EHV-1 infection of nasal mucosa explants

The equine nasal mucosa explants were inoculated with EHV-1 neurological strains 03P37, 95P105 or non-neurological strains 97P70, 94P247 and treated with RSG or QC (1, 3, 10, 30 μM), respectively. Mock inoculations and treatments were carried out in parallel. The RSG or QC treatment was performed 12 h prior to or at the same time of EHV-1 inoculation, respectively. The supernatant of cultured explants was collected at 2, 24, 48, 72 hpi for viral titration. The explants were collected and snap frozen at 0, 24, 48, 72 hpi. Cryosections were made and IF stainings were performed to analyze EHV-1 infection by confocal microscopy. In the epithelium, the plaque formation was analyzed. In the lamina propria of the mucosa, two regions of interest (ROI) were chosen for analysis of EHV-1 replication. One was the region where the epithelial cells were infected with EHV-1 (ROI_WI_) and the other was the region without EHV-1 infection in the epithelial cells (ROI_WOI_). Three independent replicates were performed for each experiment.

### The effect of RSG treatment on the distribution and numbers of CD172a^+^ cells in nasal mucosa explants infected with EHV-1

The EHV-1 infected cell types in the lamina propria of which their recruitment was affected by treatment with RSG were identified by a double IF staining. For the staining of EHV-1, the protocol described above was followed. To detect CD172a^+^ cells, CD5^+^ T lymphocytes or B lymphocytes (IgM^+^), mAbs DH59B, HT23A or 1.9/3.2 (VMRD Inc., Pullman) were used as primary antibodies and FITC-labeled goat anti-mouse IgG was used as secondary antibody. Proper controls were included for testing the specificity of the stainings. The nuclei were counterstained with Hoechst 33 342. All the stained cryosections were analyzed by confocal microscopy. Three independent replicates were performed for each experiment.

### Data analysis

Three independent experiments were performed and the data are presented as means ± standard deviations (Givens and Marley). ANOVA was used to calculate statistical significance among multiple groups. Data were classified: *P* > 0.05, not significantly different; *P* ≤ 0.05 (*), significantly different; *P* ≤ 0.01 (**), very significantly different; *P* ≤ 0.001 (***), extremely significantly different.

## Results

### The localization and quantification of CD172a^+^ cells in the equine nasal mucosa explants infected with EHV-1

**Figure 2:**
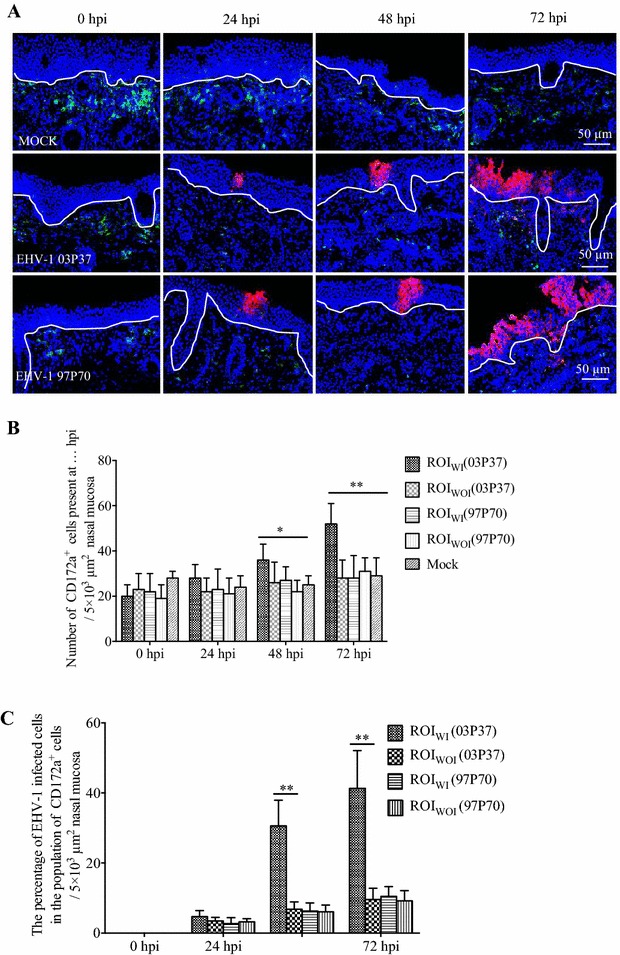
**The localization and quantification of CD172a** ^**+**^ **cells in equine nasal mucosa.** The distribution (**A**) and the number (**B**) of CD172a^+^ cells in mock inoculated and EHV-1 neurological strain 03P37 and abortigenic strain 97P70 inoculated nasal mucosa at 0, 24, 48 and 72 hpi. **C** The percentage of EHV-1 infected cells in the population of CD172a^+^ cells in the nasal mucosa. ROI_WI_ is the region including the epithelium and the lamina propria with EHV-1 infection in the epithelium whereas ROI_WOI_ is the region without EHV-1 infection in the epithelium (two-way ANOVA; *P < 0.05; **P < 0.01). EHV-1 infected cells (red); CD172a^+^ cells (green). The white line drawn on the image represents the BM. Scale bar: 50 µm.

In mock-inoculated nasal mucosa, the CD172a^+^ cells were mainly localized in the lamina propria underneath the BM. In the epithelium, the CD172a^+^ cells were present in a scattered manner (Figure 2A). Cultivation for 72 h had no impact on the distribution and number of CD172a^+^ cells. For the neurological EHV-1 strain 03P37 inoculated nasal mucosa explants, a basal to apical migration in the infected nasal mucosa area was observed. At 24 hpi, EHV-1 infected CD172a^+^ cells were observed mainly in the epithelium and less frequently underneath the BM. There was no significant difference for the number of total CD172a^+^ cells in ROI_WI_ or ROI_WOI_ compared with the mock. At 48 hpi, more EHV-1 infected CD172a^+^ cells were found in the epithelium and a few underneath the BM. Compared with the mock, the number of total CD172a^+^ cells in the ROI_WI_ was 30.5 ± 21.6% (*P* < 0.05) higher, while in the ROI_WOI_ there was no significant difference (*P* > 0.05) (Figure 2B). At 72 hpi, nearly no EHV-1 infected CD172a^+^ cells were found in the epithelium while there were much more EHV-1 infected CD172a^+^ cells observed underneath the BM. The number of total CD172a^+^ cells in the ROI_WI_ was 44.2 ± 19.5% (*P* < 0.01) higher than that in the mock (Figure 2B). The percentage of EHV-1 positive cells in the population of CD172a^+^ positive cells in the ROI_WI_ was 77.8 ± 31.5% (*P* < 0.01) higher at 48 hpi and 76.8 ± 27.9% (*P* < 0.01) higher at 72 hpi compared with the ROI_WOI_ (Figure 2C). Similar results were observed when inoculated with another EHV-1 neurological strain 95P105 (Additional file 1). For the EHV-1 non-neurological strains 97P70 and 94P247, there were no significant differences for the number and distribution of CD172a^+^ cells between ROI_WI_ or ROI_WOI_ and the mock at all time points (Figure 2; Additional file 1).

### The expression of chemokine CCL2 and CCL5 in EHV-1 infected nasal mucosa explants

**Figure 3:**
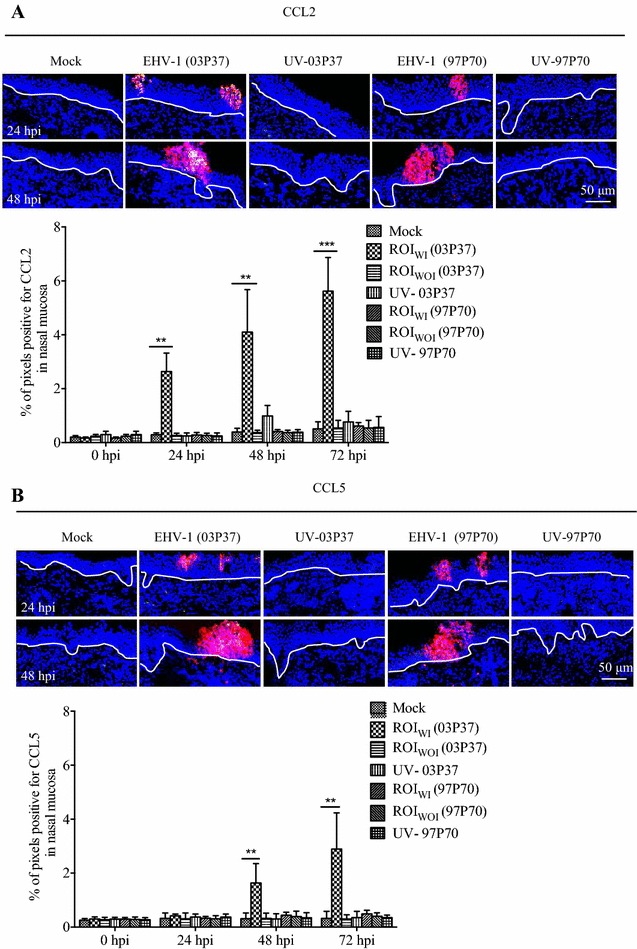
**The expression of CCL2 and CCL5 in EHV-1 infected nasal mucosa.** The expression of CCL2 (**A**) and CCL5 (**B**) in mock inoculated, EHV-1 neurological strain 03P37 and abortigenic strain 97P70 inoculated (at ROI_WI_ and ROI_WOI_) as well as their UV inactivated viruses (UV-03P37 and UV-97P70) inoculated nasal mucosa at 0, 24, 48 and 72 hpi (two-way ANOVA; **P < 0.01; ***P < 0.001). EHV-1 infected cells (red); CCL2 or CCL5 (green). The white line drawn on the image represents the BM. Scale bar: 50 µm.

Both CCL2 and CCL5 positive cells were scattered underneath the epithelial cells of mock-inoculated nasal mucosal tissues at all the time points of cultivation, whereas CXCL8 positive cells were not observed (Figures 3A and B). Upon inoculation with the EHV-1 neurological strain 03P37, the expression of CCL2 and CCL5 strongly increased and colocalized with the EHV-1 infected epithelial cells (Figures 3A and B). The total expression of CCL2 was much higher than that of CCL5. The expression of CCL2 in nasal mucosa at ROI_WI_ increased eightfold (*P* < 0.01) compared to the mock at 24 hpi, ninefold (*P* < 0.01) at 48 hpi and 11-fold (*P* < 0.001) at 72 hpi (Figure 3A). For CCL5, its expression in the ROI_WI_ of the nasal mucosa explant inoculated with EHV-1 was fourfold (*P* < 0.01) and eightfold (*P* < 0.01) higher than the mock at 48 hpi and 72 hpi, respectively (Figure 3B). There were no significant differences (*P* > 0.05) for CCL2 and CCL5 production between EHV-1 (ROI_WOI_), UV-EHV-1 and mock-inoculated explants at all the time points mentioned. The neurological strain 95P105 showed similar results as strain 03P37 (Additional file 2). For nasal mucosa inoculated with the non-neurological EHV-1 strains 97P70 and 94P247, the expression of CCL2 and CCL5 was not significantly different compared with mock-inoculated nasal mucosa (Figure 3; Additional file 2). CCL2 and CCL5 positive cells were distributed in a scattered way underneath the epithelium in the nasal mucosa in scratch-wound assay and their expression was similar with the mock (data not shown).

### Viability analysis during treatment with RSG or QC in in vitro cultures

Apoptotic cell death was detected at different concentrations (1, 3, 10, 30 μM) with RSG or QC. The number of apoptotic cells in the epithelium had a slight, but not significant increase during the 72 h cultivation period with both products. The number of apoptotic cells in the epithelium did not increase significantly for either RSG or QC (Table 1, only the data at 72 hpi were shown).

**Table 1:** **Absence of toxicity of RSG and QC in nasal mucosa explants**

Localization (nasal mucosa)	Products	Percentage (%) of TUNEL-positive cells treated at… μM at 72 hpi				
		0 (mock)	1	3	10	30
Epithelium	RSG	0.3 ± 0.2	0.4 ± 0.3	0.7 ± 0.6	0.6 ± 0.5	1.1 ± 0.6
	QC	0.4 ± 0.1	0.7 ± 0.3	1.2 ± 0.6	1.4 ± 0.6	1.6 ± 0.7
Lamina propria	RSG	0.5 ± 0.6	1.4 ± 0.8	3.0 ± 0.7	2.8 ± 1.9	4.0 ± 1.1
	QC	0.6 ± 0.2	1.2 ± 0.7	2.7 ± 0.9	3.2 ± 0.7	4.7 ± 1.6

Viability of nasal mucosa explants treated with RSG or QC at different concentrations was determined in the TUNEL assay at 72 hpi. Values are given as mean ± SD of 3 different experiments.

### The effect of RSG or QC on EHV-1 infection in nasal mucosa explants

**Figure 4:**
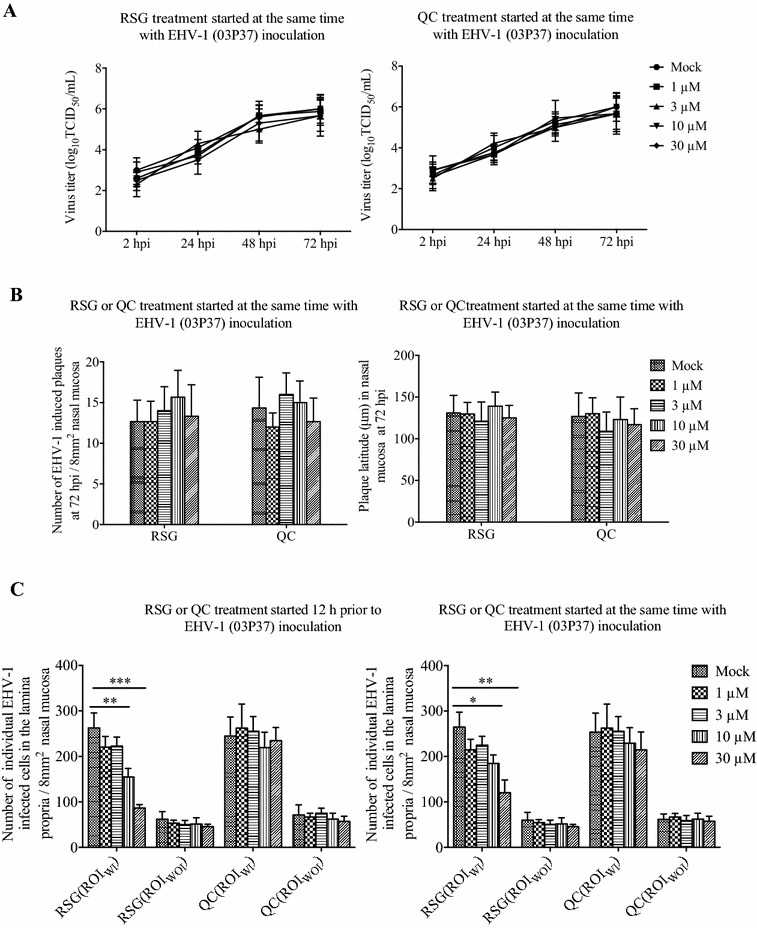
**Viral production and plaque formation of EHV-1 in equine nasal mucosa.** Viral production (**A**) at 0, 2, 24, 48 and 72 hpi and plaque formation (**B**) at 72 hpi in nasal mucosa explants inoculated with EHV-1 neurological strain 03P37 and treated at the same time with RSG or QC at different concentrations (1 μM, 3 μM, 10 μM, 30 μM). The number of individual EHV-1 infected cell in the lamina propria of nasal mucosa explants (**C**) treated with RSG or QC 12 h prior to or at the same time of EHV-1 inoculation. ROI_WI_ is the region of interest including the epithelium and the lamina propria with EHV-1 infection in the epithelium whereas ROI_WOI_ is the region of interest without EHV-1 infection in the epithelium (two-way ANOVA; **P < 0.01; ***P < 0.001)

During RSG or QC 12 h pre-treatment or treatment at the same time with the neurological EHV-1 strain 03P37 inoculation, the viral titers in the supernatant of cultured explants and the plaque formation in the epithelium were similar, so only the data of RSG or QC treatment at the same time with EHV-1 inoculation were shown. Viral titers (Figure 4A) and plaque formation among different concentrations of RSG and QC treatment did not differ significantly (Figure 4B). In the lamina propria of nasal mucosa infected with the neurological 03P37 strain, the number of EHV-1 infected cells was remarkably lower at 72 hpi when treated with RSG 12 h before inoculation or at the same time, with 41.2 ± 11.7% (*P* < 0.01) or 30.3 ± 8.2% (*P* < 0.05) at 10 μM and 67.2 ± 9.8% (*P* < 0.001) or 54.5 ± 16.8% (*P* < 0.01) at 30 μM for the ROI_WI_ (Figure 4C). This lower number of EHV-1 infected cells was not observed when treated with QC. The neurological strain 95P105 showed similar results as strain 03P37. For strain 97P70 and 94P247, pre-treatment and treatment with RSG or QC did not change virus replication in the epithelium and the number of EHV-1 infected cells in the lamina propria (Additional file 3, only the data for strain 97P70).

### The RSG treatment inhibited the migration of CD172a^+^ cells in the EHV-1 inoculated nasal mucosa explants

**Figure 5:**
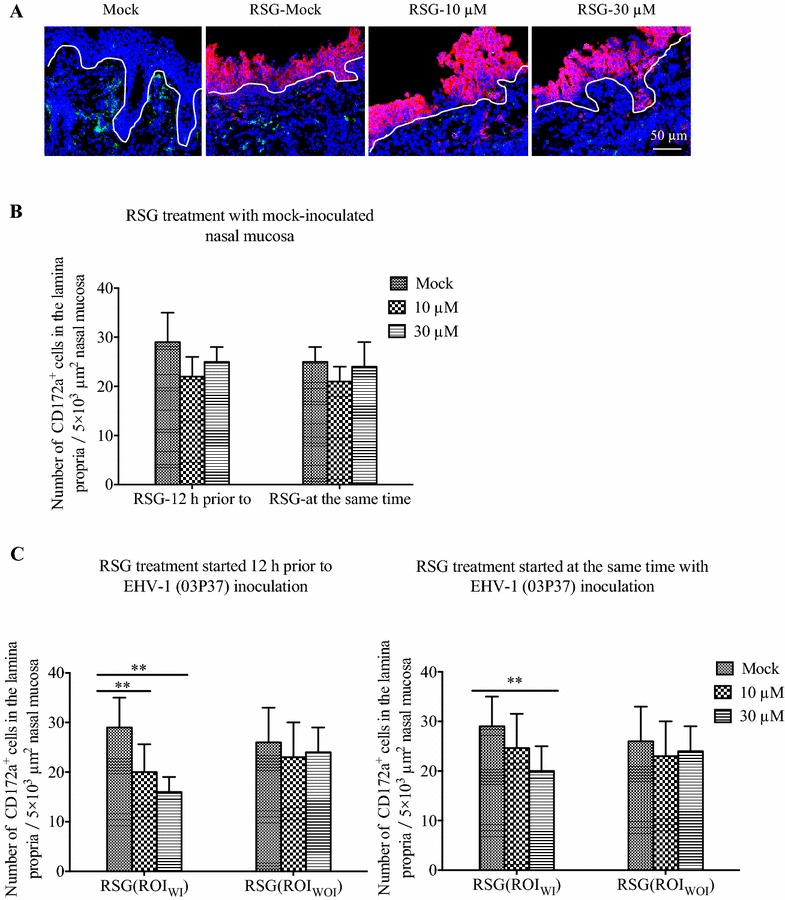
**The localization and quantification of CD172a** ^**+**^ **cells in the lamina propria treated with RSG.** The distribution (**A**) and the number of CD172a^+^ cells in the lamina propria of mock-inoculated (**B**) or EHV-1 neurological strain 03P37 inoculated (**C**) nasal mucosa explants at 72 hpi and treated with RSG 12 h prior to or at the same time of the mock or viral inoculation at a concentration of 10 μM or 30 μM. ROI_WI_ is the region of interest including the epithelium and the lamina propria with EHV-1 infection in the epithelium whereas ROI_WOI_ is the region of interest without EHV-1 infection in the epithelium (two-way ANOVA; *P < 0.05; **P < 0.01). EHV-1 infected cells (red); CD172a^+^ cells (green). The white line drawn on the image represents the BM. Scale bar: 50 µm.

In the mock-inoculated explants and EHV-1-inoculated explants, the RSG pretreatment or treatment at the same time had no effect on the number of CD5^+^ T and B-lymphocytes in the lamina propria. Within the mock-inoculated explants, the RSG pretreatment or treatment at the same time had no effect on the number of CD172a^+^ cells in the lamina propria (Figure 5B). In the EHV-1-inoculated explants, at 72 hpi, in the lamina propria of ROI_WI_ the number of total CD172a^+^ cells decreased with 28.9 ± 6.5% (*P* < 0.01) or 41.3 ± 9.6% (*P* < 0.01) at a pretreatment with 10 or 30 μM. When treated with RSG at the same time of EHV-1 inoculation, the number of total CD172a^+^ cells in the lamina propria of ROI_WI_ was reduced with 31.1 ± 7.6% (*P* < 0.01) at the concentration of 30 μM (Figure 5C). The neurological strain 95P105 showed similar results as strain 03P37 (Additional file 4).

## Discussion

Monocytic cells play an important role in the pathogenesis of EHV-1 infection [6]. CD172a^+^ monocytic cells become infected with EHV-1 in the nasal mucosa (the initial infection site), and transport the virus from the apical side of the epithelium into the deep lamina propria [11]. Our data demonstrate that a basal to apical side migration of non-infected CD172a^+^ cells was present during the early stage of the EHV-1 neurological strain infection in the epithelial cells. Indeed, at 24 and 48 hpi, CD172a^+^ cells migrated towards the infected epithelium region as fewer CD172a^+^ cells were observed underneath the BM while more infected CD172a^+^ cells were present in the epithelium at these time points. In the infected regions more CD172a^+^ cells were found at 48 and 72 hpi, most probably due to migration from deeper tissues, as there was no reduction of CD172a^+^ cells in the area lateral from the infected regions. More EHV-1 infected CD172a^+^ cells gathered in the lamina propria at 72 hpi, which indicated that after getting in contact with the virus, the infected CD172a^+^ cells migrated into the deep regions for further invasion. It has also been reported that a basal-to-apical monocyte transepithelial migration in vitro can be elicited by influenza A virus infection of primary alveolar epithelial cells and resident alveolar macrophages, which can be induced by monocyte chemoattractants CCL2 and CCL5 [14, 30]. Chemokines are secreted in response to signals such as proinflammatory cytokines where they play an important role in selectively recruiting monocytes, neutrophils, and lymphocytes. Monocyte chemoattractant protein-1 (MCP-1/CCL2) can be secreted by epithelial cells and many immune cells including monocytes and DC and is one of the key chemokines that regulate migration and infiltration of monocytes/macrophages [31, 32]. CCL5, previously known as RANTES (regulated upon activation, normal T cell expressed and secreted) is a member of the CC chemokine family. It can be produced by T lymphocytes, tumor cells and fibroblasts and recruits monocytes, T cells, basophils and eosinophils [33]. The current data in our study showed that the neurological EHV-1 infected nasal mucosa explants displayed a release of both CCL2 and CCL5. The CCL2 started to be expressed in the epithelial cells at 24 hpi, which is corresponding with the time point when the number of CD172a^+^ cells in the lamina propria decreased. This indicates that the secreted CCL2 is most probably involved in the recruitment of CD172a^+^ cells during an infection with EHV-1 neurological strains. The basal to apical migration and chemokine production were shown for neurological strains but not for the non-neurological strains. This strain specificity may be linked with the cell tropism of EHV-1. For the neurological strain 03P37, the majority of individual infected cells were CD172a^+^ cells while for the non-neurological strain 97P70, individual infected cells were equally identified as CD5^+^ T lymphocytes and CD172a^+^ cells [5]. For CD5^+^ T lymphocytes attraction, other chemokines such as, CXCL9 and CXCL10 [16, 19] may be involved. Current research is looking into the involvement of these T lymphocytic chemokines in the early pathogenesis of non-neurological EHV-1 strains. For the difference between neurological strains and non-neurological strains, there is a hypothesis that the mutation in ORF30 (DNA polymerase, A2254/G2254), which results in a modified DNA polymerase activity, enhances the virulence of this viral strain [10]. However, there is compelling evidence that this nucleotide substitution is not the only determinant for the induction of neurological disease by EHV-1 [34]. Not only ORF30, but also other ORFs may have an impact on the viral replication rate, with potential concomitant effects on neuropathogenicity [35]. This still needs to be further examined.

Absence of CCL2 and CCL5 expression in the wound-scratch assay indicated that cell death induced by wounding was not responsible for or at least, had no direct impact on their expression. CCL2 and CCL5 were not detected when inoculated with UV inactivated EHV-1. This demonstrated that the step of binding to and entry into host cells is not sufficient for the production of CCL2 and CCL5. A further stage, including viral gene expression and replication is necessary for their expression. The production of CCL2 and CCL5 was first detected at 24 hpi in the nasal mucosa infected with EHV-1. At 12 hpi, there was nothing observed (data not shown). This indicates that the chemokines CCL2 and CCL5 become expressed between 12 and 24 hpi. This is the time period during which some late viral proteins become expressed in epithelial cells and CD172a^+^ cells [36, 37]. It seems to indicate that some late viral proteins or transcription factors are involved in the induction of CCL2 and CCL5 expression. It has been reported that human cytomegalovirus (HCMV) tegument protein pp71 [38] and the viral G protein coupled receptor (vGPCR) of human herpesvirus 8 (HHV-8) lead to an increased expression of CCL2 during infection [39]. In addition, HHV-8 upregulates activating transcription factor 4 (ATF4) expression, which induces CCL2 production in endothelial cells [40]. RNase protection analyses revealed increased expression of CCL2 at 8 and 12 hpi with EHV-1 strain KyARgp2F (an EHV-1 recombinant strain from KyA, expressing the full-length gp2 protein) compared to EHV-1 strain KyA (harbors part of gp2 protein) when infecting mice [41]. It is very well possible that the full-length gp2 protein is involved in the induction of CCL2 in the early stage of infection. The difference of the time points may be explained by the fact that our detection was on the protein level, which is later than the detection of RNA transcripts. Except for gp2 protein, other EHV-1 viral proteins and transcription factors involved in the CCL2 and CCL5 induction also need to be further identified.

As we have observed the migration of CD172a^+^ cells during the EHV-1 infection in the nasal mucosa, migration inhibitors were used to treat the nasal mucosa. The treatment with RSG of equine nasal mucosa had no impact on the virus replication in the epithelium while the number of infected CD172a^+^ cells in the lamina propria decreased when treated with RSG at 10 and 30 μM. A possible explanation is that RSG treatment can inhibit the migration of CD172a^+^ cells in a dose-dependent manner during an EHV-1 infection. It showed that the migration of CD172a^+^ cells from the deeper tissues towards the infected region was inhibited in our study. This is in agreement with the research of Kintscher et al. [42], demonstrating that RSG function as peroxisome proliferator-activated receptor (PPAR) γ agonist and the activation of PPAR γ could impede DC migration from the peripheral sites of antigen capture to the DLNs. PPAR γ activation could also play a role in the spontaneous migration of intramucosal DCs and reduced migration of mice lung DCs under steady state conditions without infection [23, 43]. Our data showed that only in the condition with EHV-1 infection, the RSG treatment could inhibit the migration of CD172a^+^ cells, whereas no inhibitory impact was observed with mock-inoculation and RSG treatment. This may be due to the species specificity and the difference between in vivo and ex vivo cultures.

In conclusion, we have demonstrated that a basal to apical migration of CD172a^+^ cells in nasal mucosa was present during infections with neurological EHV-1 strains, with CCL2 and CCL5 involved in the attraction of CD172a^+^ cells towards the infected regions. RSG treatment efficiently inhibited the CD172a^+^ cells migration in a dose-dependent manner while it had no effect on the virus replication in the epithelium. A better understanding of the viral and cellular factors during this process could give new insights into the early pathogenesis of an infection with neurological EHV-1 and provide future therapeutic strategies.

## Additional files

**Additional file 1.** **The quantification of CD172a** ^**+**^ **cells in equine nasal mucosa.** (A) The number of CD172a^+^ cells in mock inoculated and EHV-1 neurological strain 95P105 and abortigenic strain 94P247 inoculated nasal mucosa at 0, 24, 48 and 72 hpi. (B) The percentage of EHV-1 infected cells in the population of CD172a^+^ cells in the nasal mucosa. ROI_WI_ is the region including the epithelium and the lamina propria with EHV-1 infection in the epithelium whereas ROI_WOI_ is the region without EHV-1 infection in the epithelium (Two-way ANOVA; *: *P* *<* 0.05; **: *P* < 0.01).

**Additional file 2.** **The expression of CCL2 and CCL5 in EHV-1 infected nasal mucosa.** The expression of CCL2 (A) and CCL5 (B) in mock inoculated, EHV-1 neurological strain 95P105 and abortigenic strain 94P247 inoculated (at ROI_WI_ and ROI_WOI_) as well as their UV inactivated viruses 95P105 and 94P247 inoculated nasal mucosa at 0, 24, 48 and 72 hpi (Two-way ANOVA; **: *P* *<* 0.01; ***: *P* < 0.001).

**Additional file 3.** **Viral production and plaque formation of EHV-1 in nasal mucosa.** Viral production (A) at 0, 2, 24, 48 and 72 hpi and plaque formation (B) at 72 hpi in nasal mucosa explants inoculated with EHV-1 abortigenic strain 97P70 and treated at the same time with RSG or QC at different concentrations (1 μM, 3 μM, 10 μM, 30 μM). The number of individual EHV-1 infected cells in the lamina propria of nasal mucosa explants (C) treated with RSG or QC 12 h prior to or at the same time of EHV-1 inoculation. ROI_WI_ is the region of interest including the epithelium and the lamina propria with EHV-1 infection in the epithelium whereas ROI_WOI_ is the region of interest without EHV-1 infection in the epithelium (Two-way ANOVA; **: *P* < 0.01; ***: *P* < 0.001).

**Additional file 4.** **The quantification of CD172a** ^**+**^ **cells in the lamina propria treated with RSG.** The number of CD172a^+^ cells in the lamina propria of EHV-1 neurological strain 95P105 inoculated nasal mucosa explants at 72hpi treated with RSG 12 h prior to (A) or at the same time (B) of the mock or viral inoculation at a concentration of 10 μM or 30 μM. ROI_WI_ is the region of interest including the epithelium and the lamina propria with EHV-1 infection in the epithelium whereas ROI_WOI_ is the region of interest without EHV-1 infection in the epithelium (Two-way ANOVA; *: *P* < 0.05; **: *P* < 0.01).
