# Supplementary material for: CCL2 and CCL5 driven attraction of CD172a+ monocytic cells during an equine herpesvirus type 1 (EHV-1) infection in equine nasal mucosa and the impact of two migration inhibitors, rosiglitazone (RSG) and quinacrine (QC)
Source: Vet Res. 2017 Feb 27;48:14. doi: 10.1186/s13567-017-0419-4 (PMC5327560; doi:10.1186/s13567-017-0419-4)
Supplement: Supplementary file 3 — Additional file 3. Viral production and plaque formation of EHV-1 in nasal mucosa. Viral production (A) at 0, 2, 24, 48 and 72 hpi and plaque formation (B) at 72 hpi in nasal mucosa explants inoculated with EHV-1 abortigenic strain 97P70 and treated at the same time with RSG or QC at different concentrations (1 μM, 3 μM, 10 μM, 30 μM). The number of individual EHV-1 infected cells in the lamina propria of nasal mucosa explants (C) treated with RSG or QC 12 h prior to or at the same time of EHV-1 inoculation. ROIWI is the region of interest including the epithelium and the lamina propria with EHV-1 infection in the epithelium whereas ROIWOI is the region of interest without EHV-1 infection in the epithelium (Two-way ANOVA; **: P < 0.01; ***: P < 0.001). [file 13567_2017_419_MOESM3_ESM.docx]

A

RSG treatment started at the same time with EHV-1 (97P70) inoculation

QC treatment started at the same time with EHV-1 (97P70) inoculation

B

RSG or QC treatment started at the same time with

EHV-1 (97P70) inoculation

RSG or QC treatment started at the same time with

EHV-1 (97P70) inoculation

C

RSG or QC treatment started 12 h prior to

EHV-1 (97P70) inoculation

RSG or QC treatment started at the same time with

EHV-1 (97P70) inoculation
