# Supplementary material for: CCL2 and CCL5 driven attraction of CD172a+ monocytic cells during an equine herpesvirus type 1 (EHV-1) infection in equine nasal mucosa and the impact of two migration inhibitors, rosiglitazone (RSG) and quinacrine (QC)
Source: Vet Res. 2017 Feb 27;48:14. doi: 10.1186/s13567-017-0419-4 (PMC5327560; doi:10.1186/s13567-017-0419-4)
Supplement: Supplementary file 4 — Additional file 4. The quantification of CD172a + cells in the lamina propria treated with RSG. The number of CD172a+ cells in the lamina propria of EHV-1 neurological strain 95P105 inoculated nasal mucosa explants at 72hpi treated with RSG 12 h prior to (A) or at the same time (B) of the mock or viral inoculation at a concentration of 10 μM or 30 μM. ROIWI is the region of interest including the epithelium and the lamina propria with EHV-1 infection in the epithelium whereas ROIWOI is the region of interest without EHV-1 infection in the epithelium (Two-way ANOVA; *: P < 0.05; **: P < 0.01). [file 13567_2017_419_MOESM4_ESM.docx]

RSG treatment started 12 h prior to

EHV-1 (95P105) inoculation

RSG treatment started at the same time with

EHV-1 (95P105) inoculation

A
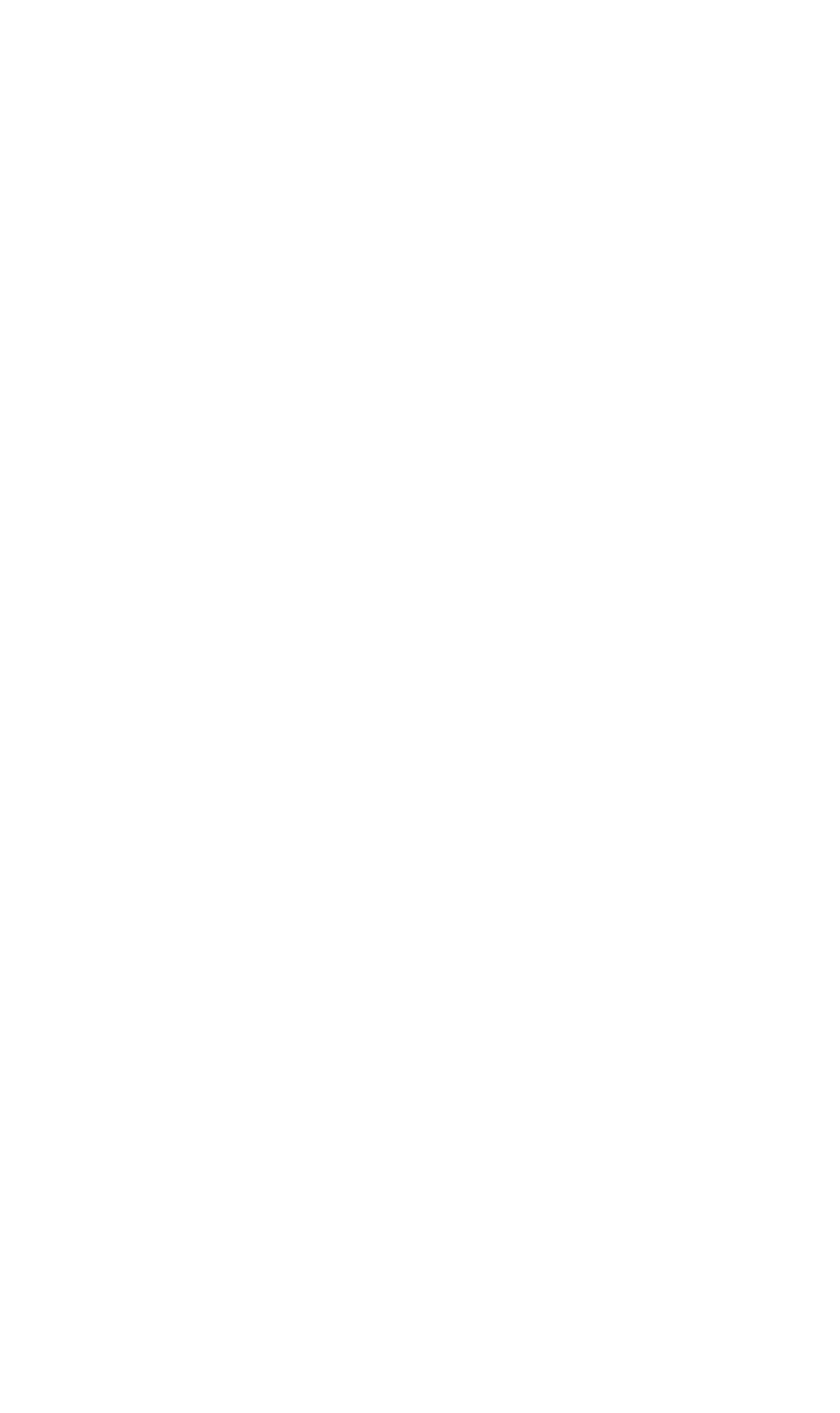


B
